# Case Report of an Empyema Identified on Lung Ultrasound

**DOI:** 10.21980/J8SH2N

**Published:** 2021-10-15

**Authors:** Michelle Brown, Carly Heffernan, Alisa Wray

**Affiliations:** * University of California, Irvine, Department of Emergency Medicine, Orange, CA

## Abstract

An empyema is a pus-filled collection within the pleural space of the thorax that most commonly results from an untreated pneumonia. Additional risk factors include poor dental hygiene, malnutrition, immunosuppression, alcohol or IV drug use, and gastroesophageal reflux. Symptoms typically include a productive cough, fever, and pleuritic chest pain, and radiographic imaging is necessary for diagnosis.^1^ In this case report, an adult male presented to the emergency department (ED) with a productive cough and shortness of breath. Ultrasound and computed tomography (CT) imaging confirmed the diagnosis of a left-sided empyema. The patient was successfully treated with a chest tube and antibiotics and was subsequently discharged eight days after admission. This case demonstrates the effectiveness of ultrasound and CT in diagnosing an empyema from other lung conditions. Ultrasound has the potential to visualize additional features not always seen on CT alone, which suggests the possibility of lung ultrasound as an alternative to a chest CT when diagnosing patients in at-risk radiation exposure groups, including children and pregnant women.

**Topics:**

Empyema, lung ultrasound, chest tube.

**Figure:**
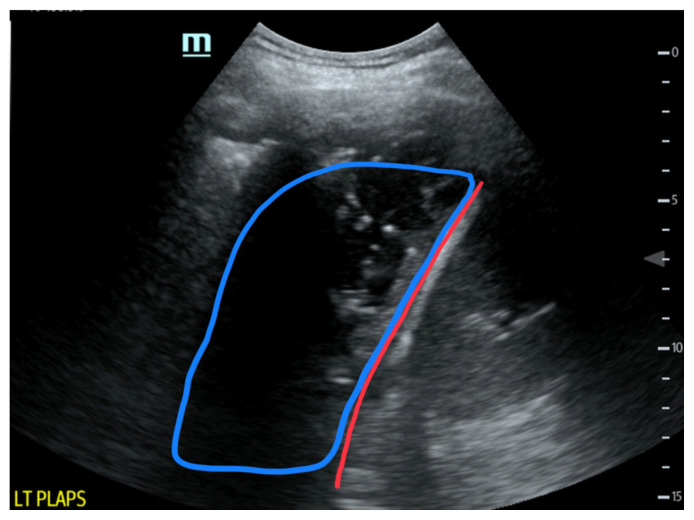


**Figure:**
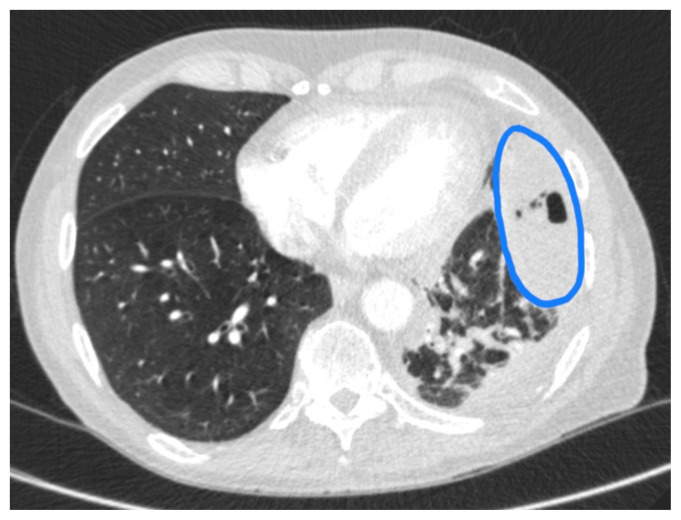


**Figure:**
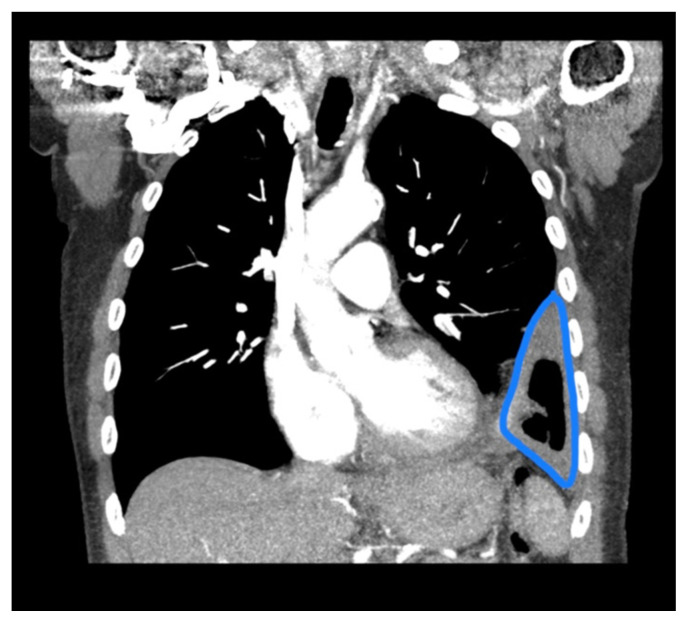


## Brief introduction

An empyema is a localized collection of pus within the pleural space.^2^ It is associated with pathogenic infection and is commonly seen as a result of untreated pneumonia.^3^ Symptoms often include malaise, fever, difficulty breathing, and weight loss, which worsen over time with delays in seeking treatment or delays in diagnosis.^4^ Radiographic hallmarks of an empyema includes the presence of lenticular-shaped fluid collections in the pleural space that displace lung parenchyma. Traditionally, computed tomography is used over chest X-ray to visualize the presence or absence of these lenticular-shaped loculated effusions.^5^ However, ultrasound is another imaging modality that can detect an empyema and is often underutilized in the emergency department (ED).^6^ This case report provides an example of a patient who was diagnosed with an empyema in the emergency department from a bedside ultrasound, and argues that ultrasound should be more heavily used in populations where radiation exposure should be minimized.

## Presenting concerns and clinical findings

In this case, a 76-year-old white male presented to the ED with progressively worsening shortness of breath and a productive yellow cough. On further discussion, he revealed a history of chronic obstructive pulmonary disease, diastolic dysfunction without heart failure, and multiple myeloma in remission. Upon medical chart review, he was recently admitted to the hospital one month prior to his current visit for hypoxia due to a pulmonary effusion and required a thoracentesis. Pleural fluid studies from that thoracentesis were consistent with an exudative effusion, and the patient was given augmentin and azithromycin for a total of ten days of treatment due to concern for a parapneumonic effusion. While at clinic for a follow up visit from that hospital stay, he complained of worsening difficulty breathing and cough, so he was sent to the emergency department for further evaluation. On initial assessment in the ED, his vital signs included a blood pressure of 144/94, a pulse of 112, a temperature of 98.2˚F, a respiratory rate of 18, and blood oxygen saturation of 98%. His physical exam was remarkable for mild tachycardia and diminished left-sided basilar lung sounds but normal respiratory effort. Given his history and concerning symptoms with tachycardia, appropriate laboratory testing and imaging were ordered, which are discussed below.

## Significant findings

Given the patient’s symptoms and findings on physical exam, the differential diagnosis included an upper respiratory infection, pneumonia, pleural effusion, empyema, COPD exacerbation, congestive heart failure, pulmonary embolism, and acute coronary syndrome, to name only a few. While awaiting the basic laboratory results and a chest X-ray in the emergency department, a bedside ultrasound was performed to assess the patient’s lungs and heart in an attempt to decrease the time to diagnosis. Using a curvilinear ultrasound probe, images of the patient were obtained from the left mix-axillary line. These images demonstrate a loculated left-sided pleural effusion (outlined in the attached ultrasound image in blue) that was moderate in size, concerning for an empyema. The diaphragm on the right (red) of the image outlines the inferior margin of the collection of pus, which is seen in the inferior aspect of the left lung. Unfortunately, rib shadows on the left side of the image prevent the full empyema from being captured in this single image. As a result of the bedside ultrasound, however, the patient was rapidly diagnosed with an empyema and was initiated on antibiotics, which is further discussed below. After his bedside ultrasound was completed, his chest x-ray revealed the expected left-sided pleural effusion. Additionally, a CT angiogram of the chest was ordered to rule out a pulmonary embolism, which was negative for an embolism but does redemonstrate the left-sided loculated pleural effusion (outlined on the CT axial and coronal images in blue).

## Patient course

As mentioned previously, the patient’s imaging modalities utilized included a chest x-ray, bedside lung ultrasound, and CT angiography of the chest. Given the results concerning for a left-sided empyema, the patient was initiated on broad spectrum antibiotics including piperacillin-tazobactam 4.5 grams (g) and vancomycin 1g while in the emergency department. Sputum cultures were ordered and the patient was admitted to internal medicine. Due to continued symptoms of shortness of breath and a productive cough during the hospitalization, interventional pulmonology was consulted for chest tube placement, which was placed on his third day in the hospital. The chest tube was placed to continuous suction and he was treated with three days of dornase alpha and alteplase for the empyema. Over the subsequent days, his sputum cultures grew gram negative rods and the piperacillin-tazobactam and vancomycin were discontinued, and the patient was scheduled on ceftriaxone 1g once per day and metronidazole 500 milligrams (mg) every eight hours for a total of eight days of antibiotics. Daily chest X-rays during his hospitalization showed improvement of his left-sided effusion and the patient’s symptoms of coughing and shortness of breath slowly resolved. Upon discharge, the patient was prescribed amoxicillin-clavulanate 872mg–125mg to be taken by mouth twice daily for three weeks. The patient was educated on the importance of seeking care early in the future for the development of any respiratory symptoms and was advised to follow-up in clinic over the next two weeks.

## Discussion

The gold standard for the diagnosis of an empyema is a CT scan of the chest, which provides an image of the gross shape of the lung cavity and exposes variations in tissue density.^9^ However, CT scans may not always capture the loculations or septations that allow for the diagnosis of an empyema over a pleural effusion.^10^ Unlike CT scans, ultrasound allows for a detailed characterization of loculations and septations within the pleural space.^10^ Using ultrasound, the pus-filled collections exhibit hyperechoic debris and thin hyperechoic septations within an anechoic effusion.^10^ Simple pleural effusions that are without infection, conversely, will appear as a region of homogenous, anechoic fluid, without any hyperechoic debris present.^11^ Furthermore, as an empyema progresses over time, the amount of hyperechoic debris and septations will increase.^12^ The amount of echogenicity, as a result, allows for a relative estimation of the severity and progression of the empyema, with more complex and progressed empyemas revealing an increased amount of hyperechoic debris on ultrasound.^11^ In our case, the combination of hyperechoic debris and septations within the pleural effusion suggests the diagnosis of a moderately developed empyema, and the CT scan was not necessary to obtain this diagnosis, but was instead necessary to rule out a pulmonary embolism.

In addition to the benefits of identifying hyperechoic structures on ultrasound, the ability of using ultrasound to diagnose an empyema serves as an opportunity to decrease radiation exposure in at-risk populations, including children and pregnant women. By avoiding CT scans and utilizing ultrasound as an effective alternative, the diagnosis can be made in a shorter amount of time while avoiding the high doses of radiation that are associated with CT imaging.^13^,^14^ Ultrasounds are also less expensive to the patient compared to the cost of a CT scan.^13^ Unfortunately, ultrasound machines may not be present in every emergency department for health care providers to use regularly, and not all providers have enough experience to feel comfortable using the machine in their daily practice to guide medical decisions.^15^ Furthermore, the larger the body habitus of a patient, the more difficult the ultrasound windows may be to obtain.^16^ An uncooperative patient also makes for a difficult ultrasound exam. In these instances where ultrasound may not be available, or if the exam is technically challenging or inconclusive, a CT scan should be used as the alternative imaging modality to make the diagnosis.

Finally, a weakness of the use of ultrasound in this instance was the choice of the probe. Ultimately, three probes may be used when examining the lungs on ultrasound, including the linear, the curvilinear, and the phased array probes.^17^ Linear probes are useful when assessing the anterior chest for the presence of lung sliding between the visceral and parietal pleura.^17^ Conversely, the curvilinear and phased array probes are better able to assess the lung parenchyma for the presence of fluid, as well as assess the lung bases along the mid-axillary lines where the lungs meet the diaphragm.^18^ In this case report, the curvilinear probe was used along the left mid-axillary line. Due to the larger footprint of the curvilinear probe, however, the probe spread over multiple rib spaces, producing an artifact known as posterior acoustic shadowing.^19^ These shadows cause a void on the ultrasound image due to the inability of the ultrasound waves to penetrate the dense ribs.^19^ As a result, the image produced has black voids over a portion of the picture, failing to fully capture the pathology seen in this patient. Of course, capturing videos of the pathology as the ultrasound operator uses the probe to fan through the thorax helps alleviate the artifact from the ribs, but perhaps a phased array probe with a smaller footprint would have allowed for better navigation between the rib spaces. A phased array probe might have eliminated some of the artifact and delivered images without any voids, permitting the measurement of the size of the empyema, which was not possible while using the curvilinear probe in this case.

In conclusion, this patient was seen in the emergency department for fever, shortness of breath, and a productive cough, and was diagnosed with an empyema using both a bedside lung ultrasound, as well as a CT angiography of the chest. Ultrasound offers the benefit of identifying hyperechoic septations and debris that characterize an empyema without the associated radiation exposure seen in CT imaging. In children and pregnant women, where radiation exposure should be minimized, an ultrasound should be used as the first imaging modality to diagnose an empyema instead of a CT scan. By implementing bedside ultrasound into the daily practice of health care providers in the emergency department, patients may receive a faster diagnosis and be spared the expense and radiation associated with CT imaging.

## Supplementary Information






