# Supplementary figures and images for: Case Report of an Empyema Identified on Lung Ultrasound
Source: J Educ Teach Emerg Med. 2021 Oct 15;6(4):V19–23. doi: 10.21980/J8SH2N (PMC10332732; doi:10.21980/J8SH2N)

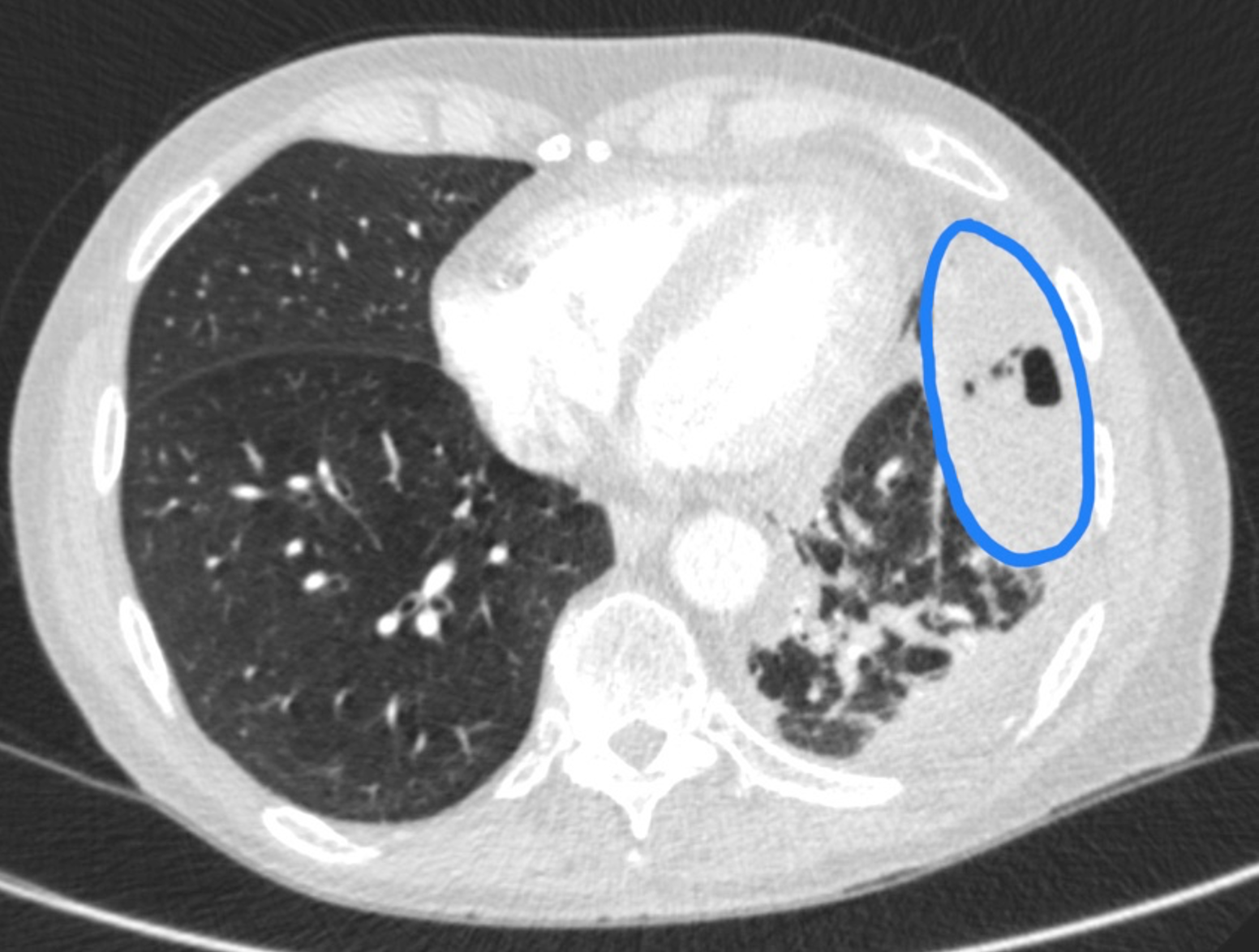

Supplement: Supplementary file 1 [file JETem-6-4-V19-supp1.jpeg]

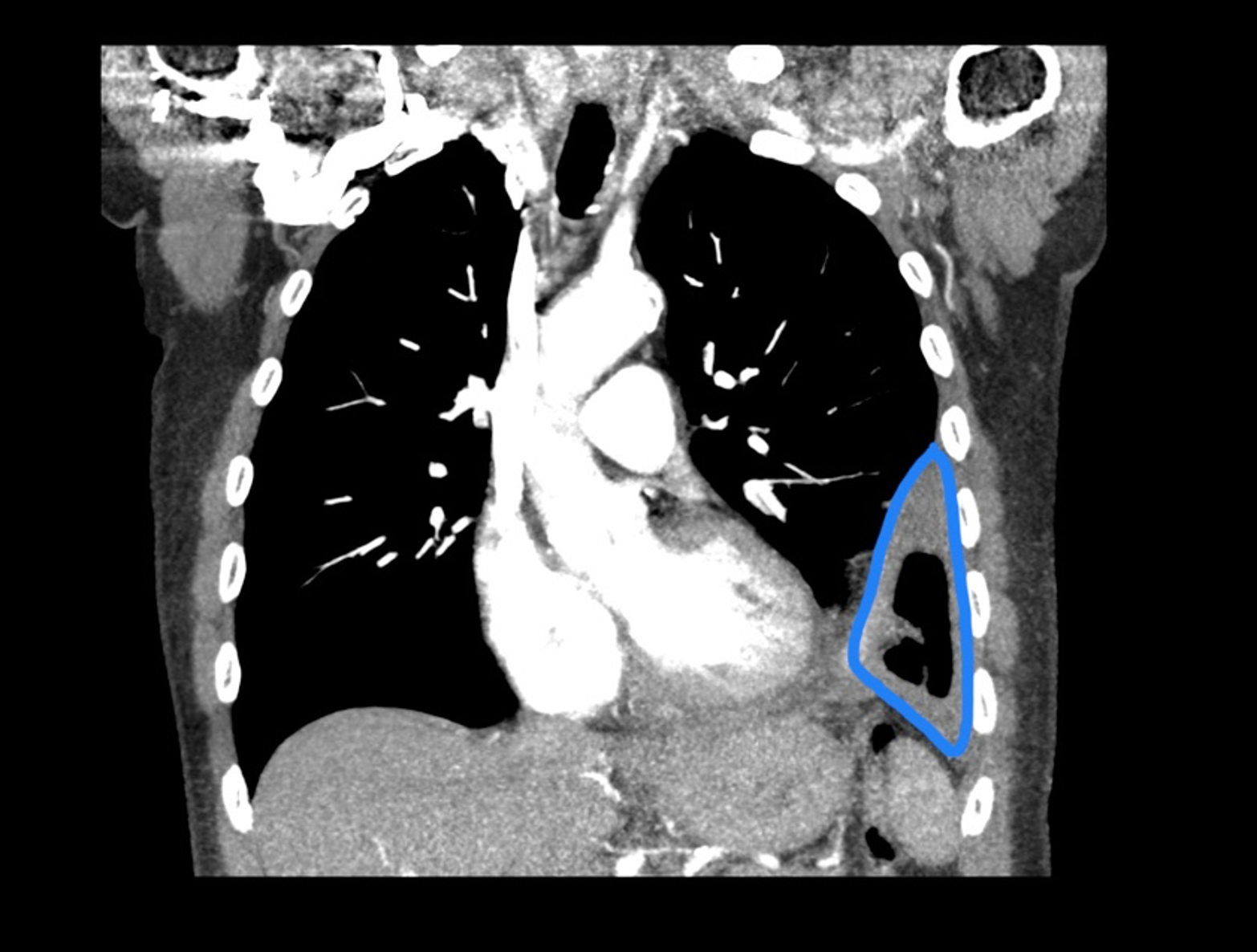

Supplement: Supplementary file 2 [file JETem-6-4-V19-supp2.jpeg]

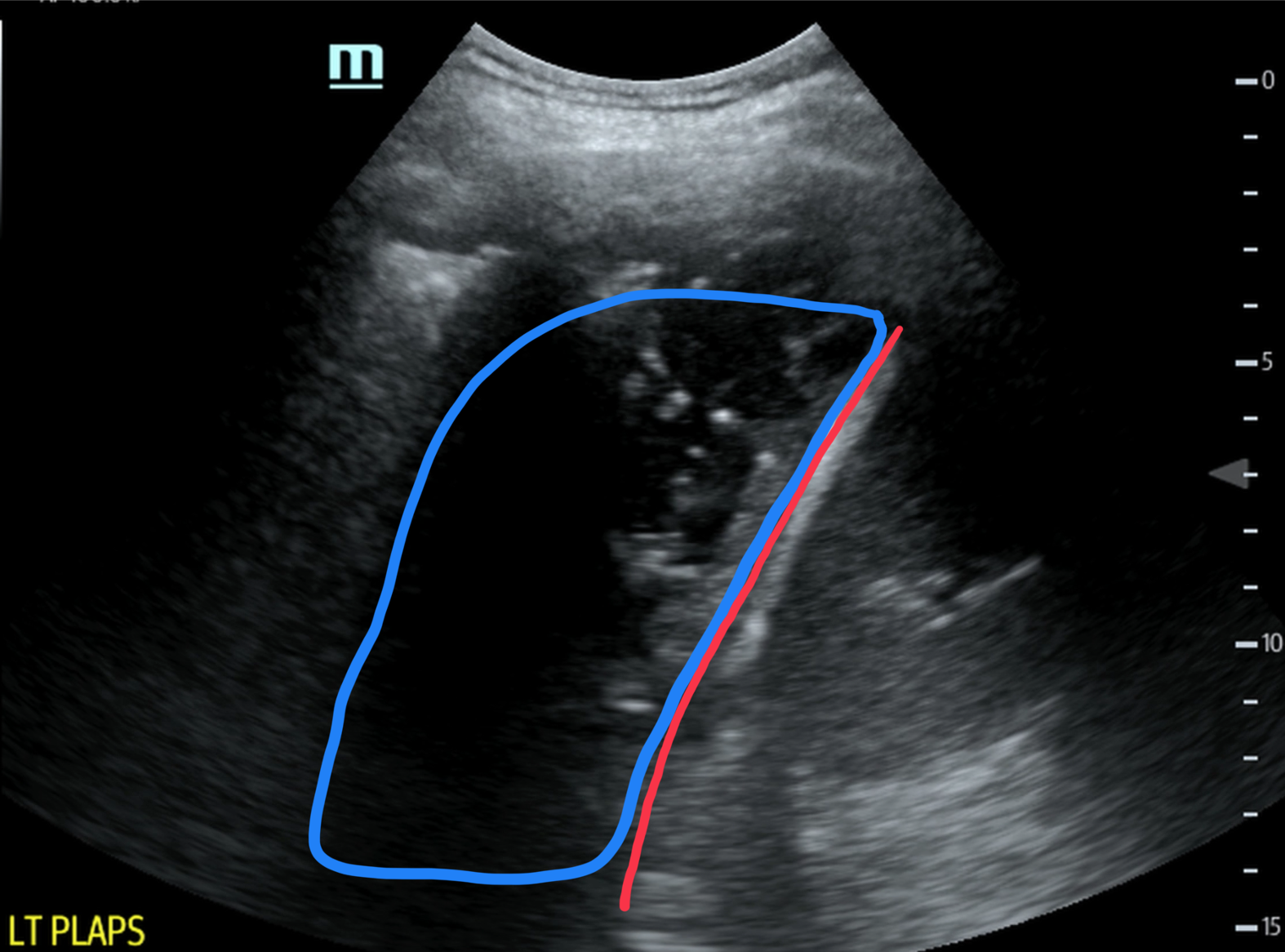

Supplement: Supplementary file 3 [file JETem-6-4-V19-supp3.jpeg]
